# Comparison of stimulus-evoked cerebral hemodynamics in the awake mouse and under a novel anesthetic regime

**DOI:** 10.1038/srep12621

**Published:** 2015-07-28

**Authors:** Paul S. Sharp, Kira Shaw, Luke Boorman, Samuel Harris, Aneurin J. Kennerley, Mimoun Azzouz, Jason Berwick

**Affiliations:** 1 Department of Psychology, University of Sheffield, Sheffield, UK; 2 Sheffield Institute for Translational Neuroscience, Department of Neuroscience, University of Sheffield, Sheffield, UK

## Abstract

Neural activity is closely followed by a localised change in cerebral blood flow, a process termed neurovascular coupling. These hemodynamic changes form the basis of contrast in functional magnetic resonance imaging (fMRI) and are used as a correlate for neural activity. Anesthesia is widely employed in animal fMRI and neurovascular studies, however anesthetics are known to profoundly affect neural and vascular physiology, particularly in mice. Therefore, we investigated the efficacy of a novel ‘modular’ anesthesia that combined injectable (fentanyl-fluanisone/midazolam) and volatile (isoflurane) anesthetics in mice. To characterize sensory-evoked cortical hemodynamic responses, we used optical imaging spectroscopy to produce functional maps of changes in tissue oxygenation and blood volume in response to mechanical whisker stimulation. Following fine-tuning of the anesthetic regime, stimulation elicited large and robust hemodynamic responses in the somatosensory cortex, characterized by fast arterial activation, increases in total and oxygenated hemoglobin, and decreases in deoxygenated hemoglobin. Overall, the magnitude and speed of evoked hemodynamic responses under anesthesia resembled those in the awake state, indicating that the novel anesthetic combination significantly minimizes the impact of anesthesia. Our findings have broad implications for both neurovascular research and longitudinal fMRI studies that increasingly require the use of genetically engineered mice.

Neurovascular coupling is a mechanism that ensures active regions of the brain are supplied with abundant levels of oxygen and glucose through a localised increase in cerebral blood flow (CBF). This active process mediated by a cascade of vasoactive signals causes an inflow of oxygenated blood which results in a decrease in the levels of deoxyhemoglobin^1^. These hemodynamic changes form the basis of the positive blood oxygenation level-dependent (BOLD) functional magnetic resonance imaging (fMRI) signal and are used as a correlate for neural activity^2^,^3^,^4^. Therefore, elucidating the physiological mechanisms underlying neurovascular coupling is an active area of research since it has a direct bearing on the analysis and interpretation of fMRI signals^5^. Moreover, with accumulating evidence that impaired neurovascular coupling is a pathogenic factor in neurodegenerative disorders, a more complete understanding of neurovascular coupling may lead to earlier differential diagnosis and novel therapeutic strategies^6^,^7^.

The use of *in vivo* animal models has been instrumental in improving our understanding of the relationship between neural and vascular responses, in particular the type of neural activity and the cellular signalling mechanisms that drive the hemodynamic response^8^,^9^. Most of these studies have been conducted in anesthetized animals due to the invasive nature of the techniques, to minimise motion artefacts and eliminate induced stress. However, anesthetics can influence neurovascular coupling at various levels by modulating neural processing, interfere directly with vascular reactivity or indirectly through systemic cardiovascular effects^10^. The degree of these effects depends upon the type of anesthetic used and the depth of anesthesia^11^,^12^,^13^. While there is still a lack of agreement on the optimal anesthetic protocols, stimulation-based paradigms in a number of species have shown preserved neurovascular coupling under anesthesia, albeit with altered hemodynamics compared to the awake condition^14^,^15^,^16^,^17^. However, neurovascular coupling investigations using anesthetized mice are far more challenging due to their sensitivity to anesthetics, which is reflected by weak and inconsistent hemodynamic responses and by the low number of studies relative to rats^12^,^18^,^19^,^20^. Since the mouse is often the animal of choice in biomedical research due the availability of genetic tools for monitoring neural activity and modelling neurodegenerative diseases, it is important to identify an anesthetic regime for neurovascular coupling studies that ensures a maximum resemblance of response to that observed in the awake state.

Therefore, in this study we measured hemodynamic responses to whisker stimulation using intrinsic optical signals in both anesthetized and awake mice. The somatosensory pathway from whiskers to the barrel cortex in rodents is a popular model for neurovascular studies since it has a well-defined topography and associated vascular system. To induce anesthesia in mice we applied a novel ‘modular‘ approach^21^, which combined injectable and volatile anesthetics to allow prolonged anesthesia for imaging, whilst limiting adverse effects on cerebrovascular physiology. To measure the hemodynamic responses, we employed two-dimensional optical imaging spectroscopy (2D-OIS), which measures changes in hemoglobin concentration and oxygenation^22^,^23^,^24^. This technique provides high spatial and temporal maps of the hemodynamic response that can be modelled to predict BOLD fMRI signals^25^,^26^ and thus inform whether the anesthetic regime can be applied to the expanding field of mouse fMRI. Finally, to establish the robustness of the hemodynamic responses under anesthesia, we compared them to those acquired in the awake state. Our major finding is that in contrast to recent reports^27^,^28^, the amplitude and speed of sensory-evoked hemodynamics in the anesthetized mouse are comparable to those measured in the awake condition.

## Results

### Cortical hemodynamic responses in the anesthetized mouse

To explore the properties of the hemodynamic response to stimuli using a novel ‘modular’ based anesthesia in mice, we applied 2D-OIS to measure functional changes in hemoglobin oxygenation and blood volume to whisker stimulation. The commonly used method for generating a stimulus-evoked hemodynamic response in the somatosensory cortex is electrical stimulation of the whisker pad^23^,^24^,^29^. Here we employed a more physiological and less noxious stimulation paradigm to negate the potential confounds of an arousal response that might be associated with electrical stimuli in mice^12^.

**Figure 1:**
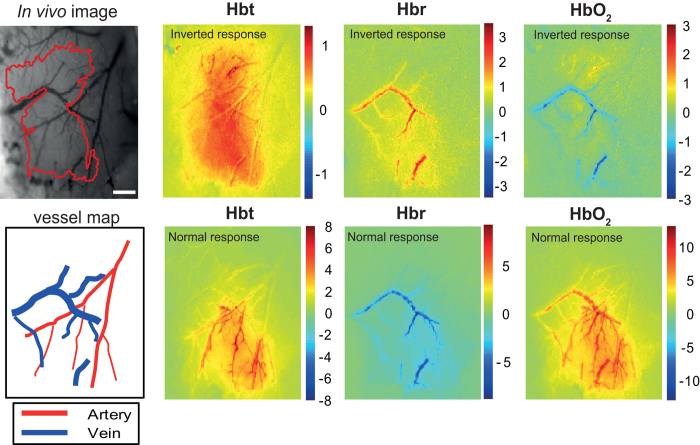
Spatial hemodynamic responses in the somatosensory cortex showing both inverted and ‘normal’ hemodynamics. Upper left panel, grayscale image to show the surface vasculature overlying the somatosensory cortex under 575 nm illumination (scale bar = 250 μM). The ‘active whisker region’ (demarcated in red), are pixels within 50% of the peak z-score response. The illustrations (lower left panel) show the location of veins and arteries. The spatial images of trial-averaged changes in concentration (μM) of oxy- (HbO_2_), deoxy- (Hbr) and total (Hbt) hemoglobin are shown for a representative mouse. Scale bar represents z-score of activated pixels. Stimulus-evoked hemodynamic responses recorded within 4 h of anesthetic induction were inverted, with an increase in Hbt associated with increased Hbr and lack of arterial recruitment. After 4 h, ‘normal’ responses were observed, with a large increase in Hbt associated with increases in HbO_2_ and clear arterial activation.

A total of 9 adult mice were used to examine the impact of anesthesia, 7 of which showed marked differences in stimulus-evoked changes in oxyhemoglobin (HbO_2_), deoxyhemoglobin (Hbr) and total hemoglobin concentration (Hbt), depending on time after the anesthetic induction (either <4 h or >4 h). Spatial maps of the averaged hemodynamic response to 16 s stimulation are presented for a representative mouse (Fig. 1). The magnitude of the response is displayed as micromolar change from baseline elicited by whisker stimulation. For the inverted response (upper panels), increases in Hbt were primarily in the parenchyma with little involvement from the arterial branches. In the draining veins, the main features were increases in Hbr and a small decrease in HbO_2_. All inverted responses occurred within 4 h of anesthetic induction. These atypical hemodynamic responses to somatosensory stimuli have been previously described in mice^30^. However, 4 h after anesthetic induction, there was a clear switch in the hemodynamic response, which is more aligned to the ‘normal’ expected changes commonly reported in rats and other species^16^,^31^,^32^. Here, we found large increases in Hbt localized to the arteries, a decrease in Hbr in the draining veins and large increase in HbO_2_ across vascular compartments.

**Figure 2:**
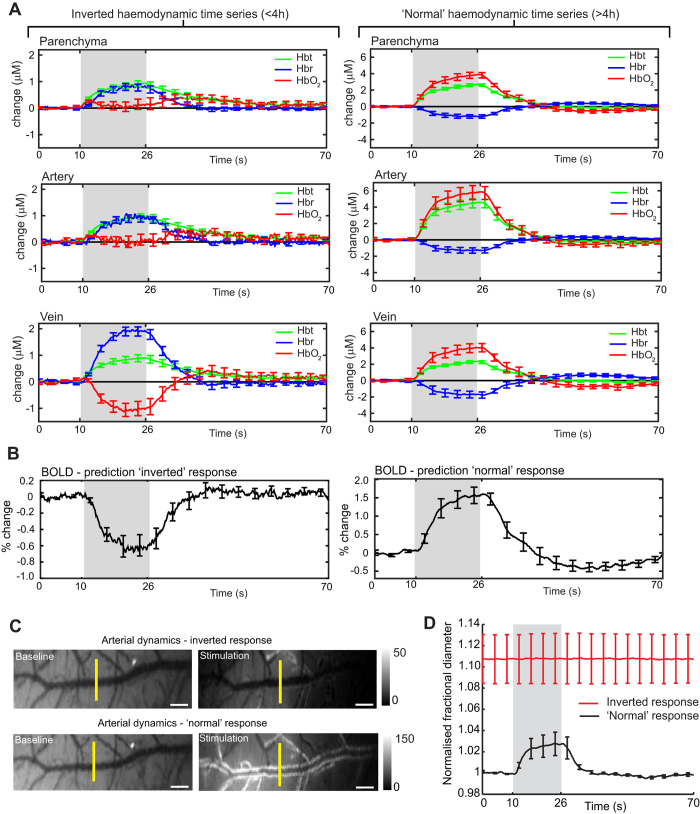
Hemodynamic time series in the anesthetized mouse. (**A**) Averaged hemodynamic time series for inverted (n = 7) and ‘normal’ (n = 9) responses, selected from different vascular compartments within the ‘active whisker region’ (parenchyma, artery, vein). Gray boxes show the duration of whisker stimulation (16 s). (**B**) Predicted blood oxygen level-dependent (BOLD) functional magnetic resonance imaging (fMRI) response using the inverted (left) and ‘normal’ (right) parenchyma time series data as input to a biophysical model^25^,^26^. (**C**) Baseline images of an artery from a representative mouse averaged for 10 s prior to stimulus and averaged for stimulus-evoked changes. The images show the artery in the same mouse for both the inverted and ‘normal’ conditions (scale bars, 100 μM). The white borders along vessel edges indicate vasodilation. (**D**) Normalised time courses of arterial diameters (full-width half-maximum) in response to stimulation for both the inverted and ‘normal’ conditions. The yellow lines, indicate the location of the diameter measurements. Data shown as mean ± s.e.m.

To assess the hemodynamic responses across all mice for both the inverted and ‘normal’ conditions, time series data were extracted from the spatiotemporal data, by selecting regions of interest from different vascular compartments (parenchyma, artery and vein) within the ‘active whisker region’ (demarcated in red, Fig. 1, upper left panel). Figure 2A shows the average time courses for both the inverted (n = 7) and ‘normal’ (n = 9) conditions. The inverted responses were characterised by small increases in Hbt across all vascular compartments, which were largely associated with increases in Hbr. The largest increases in Hbr were in the draining veins and opposed by large decreases in HbO_2_. In contrast, the ‘normal’ response profiles are dominated by a large increase in Hbt and HbO_2_, with a corresponding decrease in Hbr. This ‘normal’ or positive hemodynamic response is comparable with our previous 2D-OIS studies in adult rats^16^,^23^,^29^.

Since the blood oxygen level-dependent (BOLD) fMRI signal is predominantly driven by the decreases in Hbr^33^, we would anticipate contrasting BOLD signal changes for the inverted and ‘normal’ response conditions. Therefore, to interpret our optical imaging data for functional neuroimaging, we used the parenchymal time series data as input for a Monte Carlo simulation (MCS) of MR signal attenuation to estimate the changes in BOLD signal. It has been shown that biophysical models can be used to predict BOLD signal changes across a range of imaging field strengths^25^,^26^. We compared 7 Tesla (common field strength for *in vivo* small animal imaging) BOLD signal predictions derived from 2D-OIS data analysed with a heterogeneous tissue model^34^. For the inverted response we predicted a negative BOLD response compared to a positive BOLD response in the ‘normal’ condition (Fig. 2B).

A salient feature of the spatiotemporal characteristics between the inverted and ‘normal’ response, is the lack of arterial recruitment for the inverted condition. To explore this further, we measured arterial dynamics in response to whisker stimulation for both conditions (Fig. 2C,D). Time courses of arterial diameters (full-width half-maximum) supplying the active whisker region, confirm the artery in the inverted condition does not dilate in response to stimulation, whereas in the ‘normal’ condition there was a 2% increase in diameter. Moreover, the baseline diameters for the inverted condition were 10% greater than in the ‘normal’ condition, which suggests that the arteries were already in a dilated state, and therefore did not respond to stimulation.

### Comparing hemodynamic responses in the anesthetized and awake state

**Figure 3:**
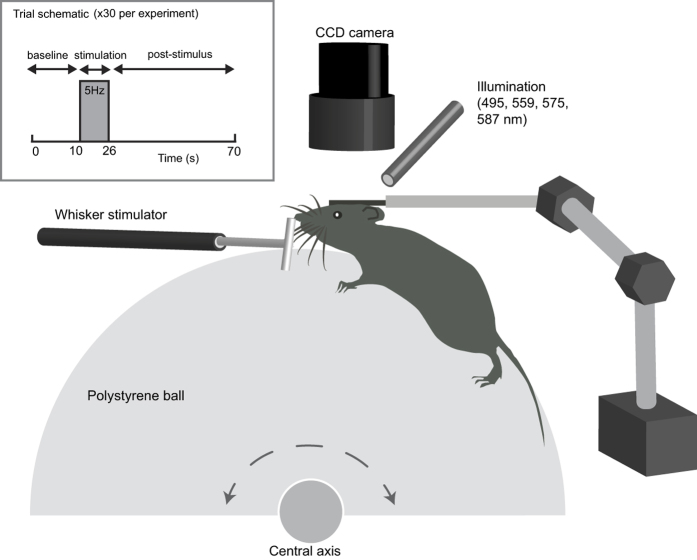
Experimental set up for awake mouse imaging. Cerebral vessels were optically imaged through a thinned-skull preparation in head fixed mice. To alleviate stress, mice were able to move on a spherical treadmill that rotates in one dimension. The schematic also shows the stimulation paradigm used to generate the hemodynamic response in the whisker barrel cortex. Whiskers were mechanically deflected at 5 Hz for 16 s and the trial was presented 30 times and averaged to create a mean trial response.

To establish an anesthetized protocol for generating robust hemodynamic responses, we first examined the effect of reducing the oxygen concentration to levels found in normal air (from 100% to ~21%) in a separate group of mice (n = 6 ). This would allow a direct comparison with mice in the awake state (see Fig. 3). In addition, to reduce the time period in which we observed the deleterious impact of anesthesia on hemodynamic responses, the initial dose of fentanyl-fluanisone, midazolam was reduced by 20% (0.8 ml/kg, i.p.). As expected, administering a lower dose of the initial anesthetic cocktail resulted in only ‘normal’ positive hemodynamics being present 2 h after induction, without the need to use higher isoflurane concentrations (0.5–0.8%) during imaging sessions. Furthermore, we found that sensory-evoked increases in Hbt within the active whisker region, appeared stable for the duration of our standard imaging session (~150 min; Supplementary Figure S1). Switching the oxygen concentration used for inhalation from 21% (medical air) to 100%, resulted in a hemoglobin baseline saturation change from 50% to 61% without a change in baseline Hbt (Supplementary Figure S2). In addition, the change in oxygen concentration also had a small but significant effect on systemic physiological parameters such as heart rate, breathing rate and arterial oxygen saturation (Supplementary Table 1).

**Figure 4:**
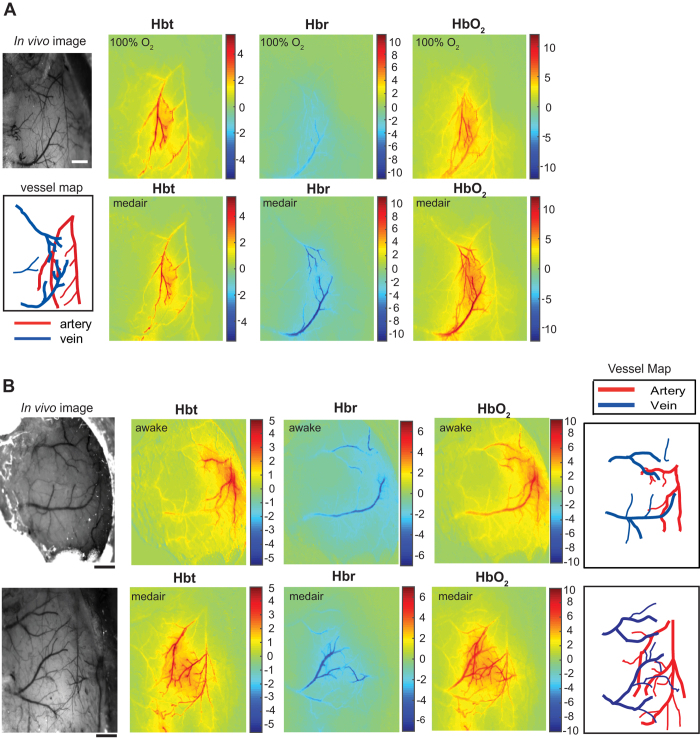
Cortical hemodynamic responses to 16 s stimulation in the anesthetized and awake mouse. (**A**) Spatial images of trial-averaged changes in concentration (μM) of oxy- (HbO_2_), deoxy- (Hbr) and total (Hbt) hemoglobin are shown for a representative anesthetized mouse under 100% O_2_ (upper panels) and the same mouse under medical air (lower panels). (**B**) Spatial images of trial-averaged changes in concentration of HbO_2_, Hbr, and Hbt, for a mouse in the awake condition (upper panels) compared to an anesthetized mouse under medical air (lower panels). Scale bars, 500 μm.

**Figure 5:**
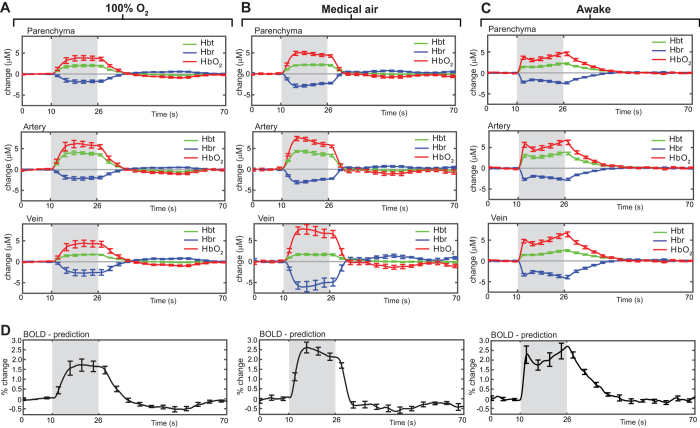
Comparing the hemodynamic response to whisker stimulation in anesthetized and awake mice. (**A**) Averaged hemodynamic time series from regions of interest selected from different vascular compartments for anesthetized mice (n = 6) under 100% oxygen and the same mice (**B**), under medical air. (**C**) Averaged hemodynamic time series from a separate group of head fixed awake mice (n = 4). (**D**) Predicted blood oxygen level-dependent (BOLD) functional magnetic resonance imaging (fMRI) response for all three conditions, using parenchyma time series data as input to a biophysical model. All data shown as mean ± s.e.m.

Spatial maps of the averaged hemodynamic response to 16 s stimulation are shown for a representative mouse under 100% oxygen or medical air (Fig. 4A). While the evoked responses in Hbt and HbO_2_ appear similar between conditions, there is a marked difference in the magnitude of the Hbr wash-out in the venous network. Averaged time series data show that in the medical air condition, stimulus-evoked responses for both Hbr and HbO_2_ across all vascular compartments were significantly larger than in the 100% oxygen condition (Fig. 5A,B; n = 6; P < 0.05, 2-tailed, paired *t*-test). However, there was no significant difference in Hbt between conditions. To interpret these optical imaging data for functional neuroimaging, parenchymal time series data was used to estimate BOLD responses. Here we found a significant increase in the amplitude of the BOLD response in the medical air condition compare to 100% oxygen (Fig. 5D, n = 6; P = 0.001, 2-tailed, paired *t*-test).

Next, we compared the hemodynamic responses in the somatosensory cortex of anesthetized mice (medical air; n = 6, as described above) and a separate group of head-fixed awake mice (see Fig. 3, n = 4). For imaging awake mice, the same experimental paradigm was used as the anesthetized group (see Fig. 3), however 5 separate experiments were conducted over 5 successive days for each awake mouse and then averaged for all experiments within each mouse.

Figure 4B shows the spatial maps of the averaged hemodynamic response to 16 s stimulation from an anesthetized and awake mouse. These maps indicate that the spatial extent, pattern and magnitude of the responses are similar under both conditions. Here we observed an increase in HbO_2_ and Hbt, and a decrease in Hbr concentration, as fresh net-oxygenated blood perfused the cortex and ‘washed out’ deoxygenated hemoglobin. Analysis of the averaged time series data, revealed that there were no significant differences in the amplitude of the hemodynamic responses between conditions (anesthetized vs awake) for all vascular compartments (P ≥ 0.05; 2-tailed, unpaired *t*-test). However, there are apparent differences in the hemodynamic response profiles between conditions. In anesthetized mice, the stimulus-evoked changes in HbO_2_ and Hbt is characterised by an initial peak and subsequent plateau followed by a return to baseline after the stimulation period (Fig. 5B). In contrast, in awake mice the response profile is more complex (Fig. 5C) Initially, the response profile is similar to that observed in anesthetized mice, however this is followed by a small partial decline to baseline before a secondary response peak, which increases in magnitude until the end of stimulation. This hemodynamic response profile is consistent with that reported in the awake rat using similar methodology^16^.

With a view to applying the anesthetized mouse preparation to BOLD fMRI, we estimated the BOLD responses using the parenchymal time series data from anesthetized (medical air) and awake mice (Fig. 5D). As expected due to similar levels of Hbr ‘wash-out’ in anesthetized (medical air) and awake mice, there was no significant difference in the peak amplitude for the predicted BOLD responses between conditions (N.S., P = 0.46, 2-tailed, unpaired *t*-test ).

**Figure 6:**
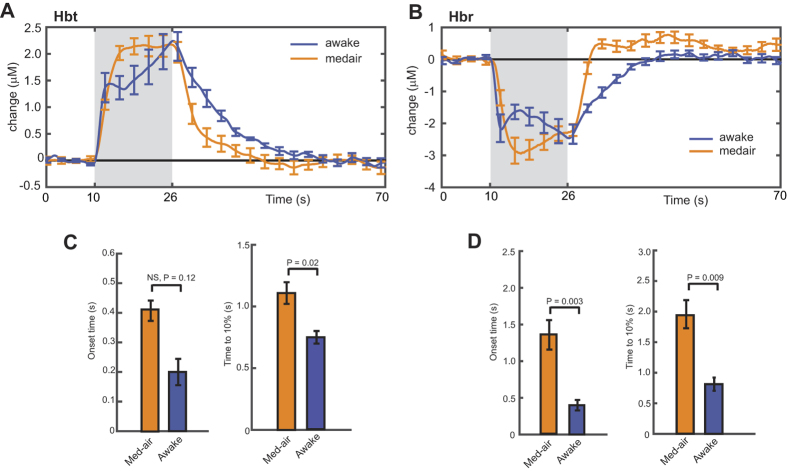
Temporal properties of the hemodynamic response to whisker stimulation in anesthetized and awake mice. (**A,B**) Summary of averaged Hbt and Hbr time series for the parenchyma region from anesthetized and awake mice. (**C,D**) Onset of the hemodynamic response and time to 10% of the first peak is shown for Hbt and Hbr. A linear line fit to the initial rising slope was used for estimating the response onset. For statistical analysis, two tailed, unpaired *t*-test (anesthetized, n = 6, vs awake n = 4). Data shown as mean ± s.e.m.

Finally, the hemodynamic parameters most relevant for predicting the BOLD response (Hbt and Hbr) were also examined for temporal characteristics. A summary of the comparison in Hbr and Hbt between anesthetized (medical air) and awake mice is shown in Fig. 6A,B. Temporal dynamics were calculated using the parenchymal time series data. The onset of the Hbt response was not significantly different between anesthetized (medical air) and awake mice (0.41 ± 0.03 s vs 0.20 ± 0.04 s, respectively; mean ± s.e.m.; Fig. 6C). However, the speed of the response, defined by the rise time to reach 10% of maximum, was slightly faster in awake mice compared to anesthetized mice (0.75 ± 0.05 s vs 1.10 ± 0.08 s, respectively; mean ± s.e.m.; Fig. 6C). The wash-out of Hbr was however, significantly faster in awake mice compared to anesthetized mice (Fig. 6D), for both onset (0.40 ± 0.07 s vs 1.35 ± 0.19 s, respectively; mean ± s.e.m.) and rise time to 10% (0.81 ± 0.11 s vs 1.94 s ± 0.23 s, respectively; mean ± s.e.m.). Nonetheless, the impact of our anesthetic regime on response dynamics appears to be far less pronounced than previous studies, which reported dramatic differences in amplitude and temporal characteristics for urethane and isoflurane anesthesia compared to the awake state^27^,^28^.

## Discussion

The present study investigated cortical hemodynamic responses to mechanical whisker stimulation in the anesthetized and awake mouse. The main finding of our study showed that by implementing a novel ‘modular’ anesthetic regime, using combined injectable and volatile anesthetics, the magnitude and speed of the hemodynamic response resembled those found in the awake state. However, the more complex temporal profile of the response in the awake state indicates that the underlying mechanisms driving the response may differ to those in the anesthetized state. In addition, our study provides a valuable understanding of baseline stimuli-evoked hemodynamics in anesthetized mice and demonstrates how suboptimal anesthesia can generate fundamentally different hemodynamic responses.

Neurovascular coupling studies are widely conducted in anesthetized animals to limit motion artefacts and avoid stress. However, anesthetics can profoundly affect the neurovascular response due to broad actions on neural processing, vascular reactivity and baseline physiology^10^. This is particularly evident in mice, which show poor reproducibility and weaker neurovascular responses compared to rats and other species^12^,^18^,^27^,^28^,^30^,^35^,^36^. To characterize stimulus-evoked hemodynamics we used two-dimensional optical imaging spectroscopy (2D-OIS), which measures changes in hemoglobin concentration and oxygenation. This technique has been central to improving our understanding of vascular events underlying neurovascular coupling^22^,^23^,^24^,^29^,^31^,^37^,^38^. Furthermore, since the BOLD fMRI response is closely linked to the concentration of Hbr, the hemodynamic data generated by 2D-OIS can offer a meaningful insight into the application of this anesthetic regime for mouse fMRI studies. Notably, there has only been one report using 2D-OIS in mice, which showed unique or inverted hemodynamic changes compared to the rat and other species; where Hbr and Hbt increased, and HbO_2_ decreased^30^. Our findings are in part, consistent with this report, since we observed similar inverted responses. However, this was only a transient feature, as a ‘normal’ response profile (decrease in Hbr an increase in HbO_2_ and Hbt) was present 4 h after the initial anesthetic induction, showing similar spatiotemporal characteristics to those reported in rats. The inverted response previously observed in mice^30^ was suggested to be a result of a disproportionately high brain capillary density, with functional hyper-perfusion being less than oxygen consumption. Since we have shown a switch to a ‘normal’ hemodynamic response in all mice, a more likely explanation is that the inverted response is a product of anesthetic-induced interference. We show that during the inverted response, the middle cerebral artery fails to respond to sensory-stimuli, as opposed to prominent arterial activation during the ‘normal’ response. The lack of arterial contribution, either suggests the mechanism linking capillary dilation to surface cerebral vessels was affected or that the surface arteries were already maximally dilated. Our analysis of arterial dynamics favours the latter, since the artery is more dilated prior to stimuli presentation for the inverted response compared to the ‘normal’ response. Furthermore, stimulus-evoked changes in arterial diameter only occurred during the ‘normal’ response condition.

Our anesthetic regime was chosen to take advantage of the synergistic interactions between injectable (fentanyl-fluanisone, midazolam) and volatile anesthetics (isoflurane), which allows the dose of each component to be reduced, while enabling sufficient anesthesia. This approach is widely used in human and veterinary medicine but rarely in mice^21^,^39^,^40^. A salient feature of fentanyl-fluanisone, midazolam anesthesia which inspired its use for imaging studies is the prolonged sedative state (>6 h) that follows the short-term surgical depth anesthesia (40 mins). Supplementing the deep sedative state with low levels of isoflurane (0.5–0.8%), induces stable anesthesia for a considerable period (>8 h). The inverted response observed in the Prakash *et al.*^30^ study was potentially caused by the vasodilatory effects of halothane administered at 1–2%. In contrast, being able to use a lower dose of isoflurane (0.5–0.8%) in our preparation, does not appear to significantly modulate ‘normal’ neurovascular coupling^19^,^36^,^41^. The initial inverted responses that we observed are presumably a result of the short-acting and fast metabolized injectable anesthetic known to cause vasodilation^42^. Reducing the dose of the injectable anesthetic, shortened the period of inverted responses without requiring a higher concentration of isoflurane to maintain anesthesia for imaging experiments.

To further validate our anesthetic regime, we compared the hemodynamic responses in anesthetized (medical air) and awake mice. Remarkably, we found no significant differences in the amplitude of the hemodynamic response between conditions. In addition, the onset time of the response measured by total blood volume changes in the parenchymal compartment was also equivalent. There was however, a small but significant attenuation in the rise time of the response. Significantly, the temporal profile of the response in the awake mouse was also more complex. Here, the most prominent difference between the hemodynamic response profile under the anesthetised and awake conditions was the contrasting magnitude of the initial peak (large vs small, respectively), which is in agreement with other studies in mice and rats using a long duration stimulation paradigm^16^,^27^. Thus, while our data suggest that vascular reactivity is largely preserved under our anesthetic regime, it is possible that subtle differences in the neuronal response to stimulation between both conditions, such as reduced sensory adaptation during arousal^43^ may contribute to the more complex hemodynamic response observed in the awake state. Whilst technically very challenging, further research involving concurrent recordings of neural activity and hemodynamic responses in anaesthetized and awake mice will be required to elucidate the impact of anesthesia on the mechanisms underlying neurovascular coupling. To our knowledge there have only been two studies that directly compared the hemodynamic responses in anesthetized and awake mice^27^,^28^. These studies describe profound effects of urethane and isoflurane (1–2%) anesthesia on response amplitude and dynamics, which were attributed to an effect on neurovascular mechanisms rather than underlying neural activity. Furthermore, in rats, in which control of baseline physiology is relatively less challenging, anesthesia was also shown to considerably weaken the neurovascular response compared to the awake state^15^,^16^,^17^. The findings of the present study demonstrate that by introducing a ‘modular’ or ‘balanced’ anesthetic regime, it is possible to effectively minimize the impact of anesthesia on the mechanisms underlying neurovascular coupling in mice.

From a technical perspective, the use of 2D-OIS offers distinct advantages over other imaging modalities, especially in relation to assessing baseline vascular network hemodynamics. The most common technique used for assessing cerebrovascular function in mice is laser Doppler flowmetry (LDF) which measures dynamic changes of cerebral blood flow (CBF) in the microvasculature but suffers from low spatial resolution^44^,^45^,^46^. More powerful methods such as 2-photon microscopy (TPM) offer high-resolution imaging of the cortical microvasculature and measurements of red-blood cell velocities^27^,^47^,^48^,^49^. However, assessing the hemodynamic response to stimulation across the surface vascular network is not practical using TPM. Recently, functional ultrasound imaging has been shown to be a valuable addition to these techniques and is able to measure cerebral blood volume (CBV) and CBF at a better spatiotemporal resolution^50^. Similarly, 2D-OIS also provides high spatial and temporal maps of cerebral blood volume and oxygenation changes. This enabled us to investigate the evolution of the hemodynamic responses within parenchymal, arterial and venous compartments. To illustrate these advantages, our 2D-OIS data revealed that during the inverted response caused by profound anesthetic effects, Hbt increased in the parenchymal compartment. Using LDF or TPM to measure changes in CBF in the microvasculature may incorrectly assume a positive hemodynamic response to stimulation. Moreover, TPM is increasingly being employed to elucidate the underlying mechanisms driving the neurovascular response by imaging blood flow changes concurrently with cell-specific markers of activity^51^,^52^,^53^,^54^. An important focus is on the role astrocytes in mediating the neurovascular response but many of these studies have produced conflicting data^51^,^55^,^56^,^57^, which in part may be attributable to poor anesthesia and a departure from appropriate physiological conditions required for a stable hemodynamic response. Our study provides a valuable reference of baseline hemodynamic responses in the anesthetized mouse, which will support future neurovascular coupling studies in mice.

Finally, our study also has important implications for establishing reliable fMRI BOLD responses in mice, which to date have been markedly inconsistent^12^,^18^,^19^,^35^,^36^. Again, since the BOLD response is particularly sensitive to the modulatory effects imposed by anesthesia, research into defining an optimal anesthetic regime is constantly evolving. Modelling our hemodynamic response data from the parenchymal compartment of anesthetized mice, we predicted a 3% BOLD signal change following whisker stimulation, which did not differ significantly from that in the awake state. Providing sufficient depth of anesthesia in mice while limiting signal attenuation is particular challenging in fMRI imaging, since the commonly used peripheral electrical stimulation often results in an arousal response and may mask focal activation^12^,^58^. Our experimental approach of using mechanical whisker deflection while maintaining a robust activation may negate this problem. In the future it will be interesting to apply both our novel anesthetic regime and stimulation protocol to discover its validity in longitudinal mouse fMRI studies.

## Methods

### Anesthesia and cranial window surgery

All animal procedures were performed in accordance with the guidelines and regulations of the UK Government, Animals (Scientific Procedures) Act 1986, the European directive 2010/63/EU, and approved by the University of Sheffield Ethical review and licensing committee. Adult female C57BL/6 mice (21–25 g) were anesthetized with fentanyl-fluanisone (Hypnorm, Vetapharm Ltd), midazolam (Hypnovel, Roche Ltd) and water (1:1:2 by volume; 1.0 ml/kg, i.p.) for surgery and maintained using isoflurane (0.5–0.8%) in 100% oxygen. A homoeothermic blanket (Harvard Apparatus) maintained rectal temperature at 37 ˚C and a mouse pulse oximeter (MouseOx Plus; Starr Life Sciences Corp.), monitored heart rat, breathing rate and arterial oxygen saturation.

Mice were placed in a stereotaxic frame (Kopf Instruments) and bone overlying the right somatosensory cortex was thinned to translucency with a dental drill to form an optical window (~3 mm^2^). A thin layer of clear cyanoacrylate cement was applied to reinforce and smooth the window and reduce specular reflections from the skull surface during imaging. Approximately 1 h was allowed between the completion of surgery and starting the imaging experiments to reduce any acute effects of the surgical procedure. For imaging mice under anesthesia, the first group of mice (n = 9), were anesthetized as stated above and maintained under 0.5–0.8% isoflurane in 100% oxygen. A second group of mice (n = 6) were anesthetized with a lower dose of hynorm/hynovel (0.8 ml/kg, i.p.), whilst using the same levels of isoflurane (0.5–0.8%). In addition, for the imaging experiments, each mouse was maintained under isoflurane delivered in either 100%, which then was then switched to medical air (~21% O_2_) or vice-versa. The order in which this occurred was randomly assigned.

### Awake imaging

A reinforced optical window was formed in adult female mice (n = 4) as described above. In addition, the eyes were protected using viscotears® (Novartis) during surgical preparation. A stainless steel head plate with a 5 mm diameter hole was secured to the skull using dental cement^28^ (see Fig. 3; Super bond C&B; Sun Medical). The mice were housed for 1-week to recover from surgery. Next, the mice were acclimatized to the experimenter, the imaging room and finally to head-fixation on the spherical treadmill^59^ (Styrofoam ball, 20 cm diameter). This involved daily training sessions. During the first session (~10 min), mice were handled by the experimenter in the imaging room, allowing movement from hand to hand. The mice were also allowed to explore the Styrofoam ball for ~10 min, without head fixation, while the handler manually controlled the ball’s rotation. The second session, consisted of repeating the format of the first session. The third session began with head-fixing the mice on the ball with the room lights on (10 min). The lights were turned off and acclimatization continued for another 20 min. These sessions were repeated daily (30 min) with the lights off until the mice learnt to move freely on the ball and showed signs of natural grooming behaviour when stationary (usually 2–3 sessions). A sweet reward was also given after every training session (a piece of toffee popcorn, Sunkist). For the final two training sessions (30 min) the whisker stimulator was introduced for the entire training session and whiskers mechanically deflected for 16 s in every 70 s.

### Mechanical whisker stimulation

Whiskers were mechanically deflected at 5 Hz for 16 s using a plastic T-bar attached to a stepper motor. Whiskers were deflected ~1 cm in the rostro-caudal direction. To improve signal-to-noise, each experiment consisted of 30 stimulus presentation trials of 70 s duration (Fig. 3). Recorded imaging data were subjected to spectral analysis described below before being averaged to create a mean trial.

### 2-Dimensional optical imaging spectroscopy (2D-OIS)

2D-OIS was used to estimate changes in cortical oxyhemoglobin (HbO2), deoxyhemoglobin (Hbr) and total hemoglobin concentration (Hbt). To generate spatial maps of hemodynamic responses, the cortex was illuminated with 4 wavelengths of light (495 ± 31 nm, 559 ± 16 nm, 575 ± 14 nm, and 587 ± 9 nm) using a Lambda DG-4 high-speed galvanometer (Sutter Instrument Company, USA). Remitted light was collected using a Dalsa 1M60 CCD camera at 184 × 184 pixels (resolution ~75 μm), with a frame rate of the 32 Hz, and synchronised to filter switching, giving an effective frame rate of 8 Hz. The spectral analysis was based upon the path length scaling algorithm (PLSA) described previously^23^. Briefly, the algorithm uses a modified Beer-Lambert Law with a path length correction factor. We estimated the concentration of hemoglobin in tissue at 104 μM based on previous measurements^60^ and saturation estimated to be 50% when breathing normal air. Under 100% O_2_, we estimated the saturation to be 61% (Supplementary Figure S2). The spectral analysis produced 2D images over time of HbO_2_, Hbr, and Hbt. The depth sensitivity of this technique has been investigated with further Monte Carlo stimulation and has been reported previously^23^,^60^.

### Selection of regions of interest (ROI) for 2D-OIS data

To select regions of interest for subsequent time-series analysis, Hbt changes evoked by whisker stimuli were analysed using a general linear model (GLM) statistical parameter mapping (SPM) approach^61^. The time series for each pixel was regressed against a design matrix of a representative ‘box-car’ hemodynamic response function with a ramp and DC offset. Subsequent ‘activation’ z-scores were calculated on a pixel-by-pixel basis. All pixels within 50% of the maximum z-score were selected and formed the ‘active whisker region’ chosen for time-series analysis. Arterial, parenchyma and vein ‘sub-regions’ were then selected within the ‘active whisker region’. Raw image data is presented, time series data was smoothed using a Savitzky-Golay filter to remove heartbeat and breathing artefacts.

### Statistical analysis

The peak amplitude of the hemodynamic response (HbO_2_, Hbr and Hbt) during a 16 s stimulation for different vascular compartments (artery, vein, and parenchyma) was calculated as the maximum change in hemoglobin concentration (μm) relative to baseline (± s.e.m., standard error of the mean). All statistical analyses were performed in MATLAB® (MathWorks), and the maximum response was compared across groups using a 2-tailed paired or unpaired Student’s *t*-test as appropriate. Two independent factors were tested either the oxygen concentration (100% O_2_ vs medair) or the state (anesthetized vs awake). To calculate stimulus ‘onset time’, two points were chosen at 20% and 70% of the maximum response and a line of best fit was drawn. The intercept of this line with the x-axis (y = 0) determined the time at which the response began. A Student’s t-test was used to compare across conditions (anesthetized vs awake). The significance level was set at P < 0.05.

## Additional Information

**How to cite this article**: Sharp, P. S. *et al.* Comparison of stimulus-evoked cerebral hemodynamics in the awake mouse and under a novel anesthetic regime. *Sci. Rep.* **5**, 12621; doi: 10.1038/srep12621 (2015).

## Supplementary Material

Supplementary Information
