# Corrigendum: Comparison of stimulus-evoked cerebral hemodynamics in the awake mouse and under a novel anesthetic regime

**DOI:** 10.1038/srep14890

**Published:** 2015-10-08

**Authors:** Paul S. Sharp, Kira Shaw, Luke Boorman, Samuel Harris, Aneurin J. Kennerley, Mimoun Azzouz, Jason Berwick

Scientific Reports 5: Article number: 12621; 10.1038/srep12621 published online: 07 28 2015; updated: 10 08 2015.

This Article contains typographical errors.

In the Results section under subheading ‘Comparing hemodynamic responses in the anesthetized and awake state’,

“In addition, to reduce the time period in which we observed the deleterious impact of anesthesia on hemodynamic responses, the initial dose of fentanyl-fluanisone, midazolam was reduced by 20% (0.8 ml/kg, i.p.).”

should read:

“In addition, to reduce the time period in which we observed the deleterious impact of anesthesia on hemodynamic responses, the initial dose of fentanyl-fluanisone, midazolam was reduced by 20% (8.0 ml/kg, i.p.).”

In the Methods section under subheading ‘Anesthesia and cranial window surgery’,

“Adult female C57BL/6 mice (21–25 g) were anesthetized with fentanyl-fluanisone (Hypnorm, Vetapharm Ltd), midazolam (Hypnovel, Roche Ltd) and water (1:1:2 by volume; 1.0 ml/kg, i.p.) for surgery and maintained using isoflurane (0.5–0.8%) in 100% oxygen.”

should read:

“Adult female C57BL/6 mice (21–25 g) were anesthetized with fentanyl-fluanisone (Hypnorm, Vetapharm Ltd), midazolam (Hypnovel, Roche Ltd) and water (1:1:2 by volume; 10.0 ml/kg, i.p.) for surgery and maintained using isoflurane (0.5–0.8%) in 100% oxygen.”

In addition,

“A second group of mice (n = 6) were anesthetized with a lower dose of hynorm/hynovel (0.8 ml/kg, i.p.), whilst using the same levels of isoflurane (0.5–0.8%).”

should read:

“A second group of mice (n = 6) were anesthetized with a lower dose of hynorm/hynovel (8.0 ml/kg, i.p.), whilst using the same levels of isoflurane (0.5–0.8%).”

