# Supplementary material for: Comparison of stimulus-evoked cerebral hemodynamics in the awake mouse and under a novel anesthetic regime
Source: Sci Rep. 2015 Jul 28;5:12621. doi: 10.1038/srep12621 (PMC4517464; doi:10.1038/srep12621)
Supplement: Supplementary Information [file srep12621-s1.pdf]

Supplementary Information

Comparison of stimulus-evoked cerebral hemodynamics in the awake mouse and under a novel anesthetic regime

Paul S Sharp[1,2]*, Kira Shaw[1], Luke Boorman[1], Samuel Harris[1], Aneurin J Kennerley[1], Mimoun Azzouz[2], Jason Berwick[1].

[1]Department of Psychology, University of Sheffield, Sheffield, UK
[2]Sheffield Institute for Translational Neuroscience, Department of Neuroscience, University of Sheffield, Sheffield, UK

*Correspondence: Dr P S Sharp. E-mail: p.sharp@sheffield.ac.uk

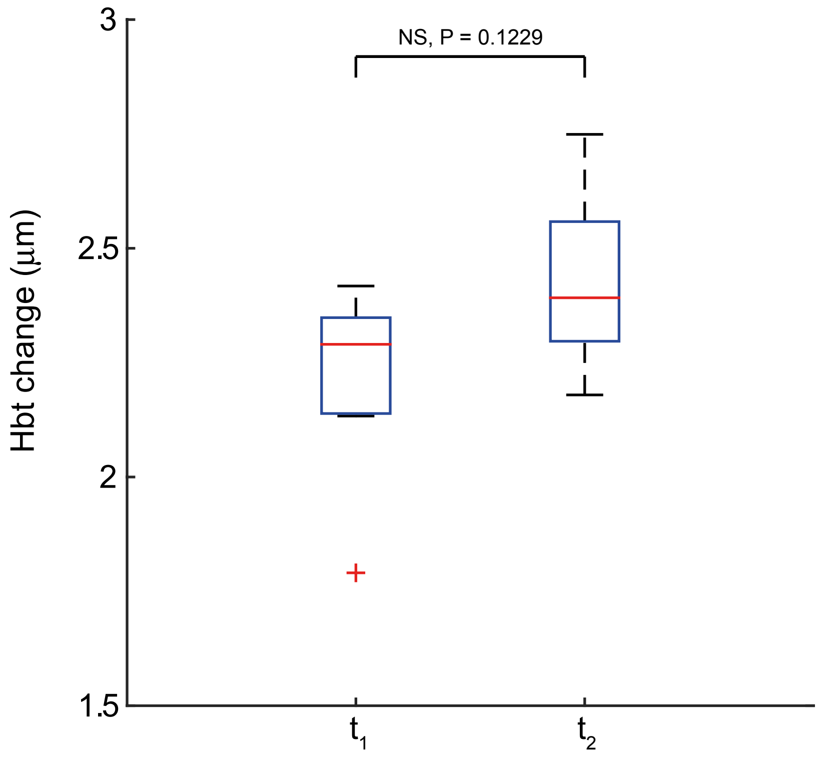

Supplementary Figure 1. Box plots comparing the average Hbt response (median and interquartile range) in the active region during a 16 s whisker stimulation (5 Hz) at t_1 (0-35 min) and at t_2 (150 - 185 min). All mice (n = 6) were under fentanyl-fluanisone/midazolam and isoflurane anesthesia (100% O_2). NS, non-significant difference between t_1 and t_2; 2-tailed paired *t*-test.

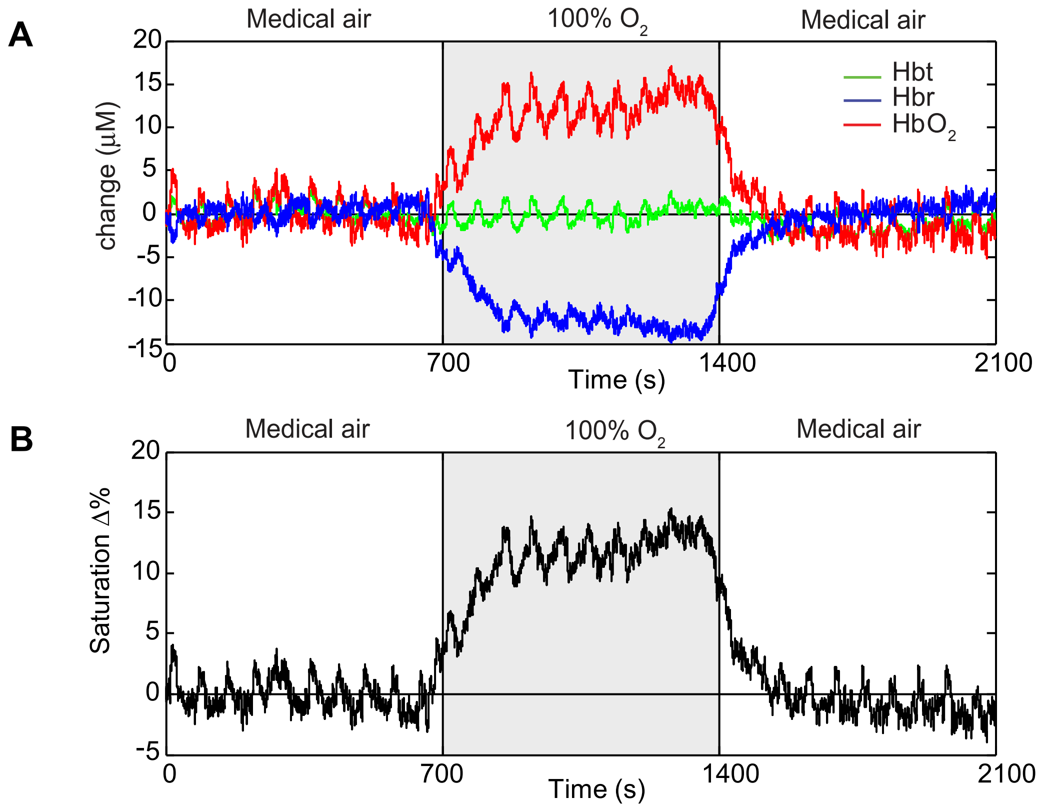

Supplementary Figure 2. The effect of medical air on baseline hemodynamics. **(A)** Averaged time series (n = 4) for Hbt, HbO_2, Hbr. **(B)** Percentage change in hemoglobin saturation caused by increasing the levels of oxygen from 21% (medical air) to 100%.

O_2 Saturation [%]		Heart rate [bpm]		Breathing rate [bpm]	
100% O_2	Medical air	100% O_2	Medical air	100% O_2	Medical air
**98.4** *± 0.1*	**96.4***** *± 0.3*	**405.4** *± 9.0*	**427.6*** *± 10.2*	**91.6** *± 5.0*	**108.0**** *± 3.2*

Supplementary Table 1. The effect of medical air on systemic physiological parameters. All data are mean ± SEM (n = 5). Significant differences, 2-tailed, paired *t*-test, *P < 0.04, **P < 0.02, ***P < 0.002.
